# Microbial Communities in Sediments of Lagos Lagoon, Nigeria: Elucidation of Community Structure and Potential Impacts of Contamination by Municipal and Industrial Wastes

**DOI:** 10.3389/fmicb.2016.01213

**Published:** 2016-08-05

**Authors:** Chioma C. Obi, Sunday A. Adebusoye, Esther O. Ugoji, Mathew O. Ilori, Olukayode O. Amund, William J. Hickey

**Affiliations:** ^1^Department of Microbiology, University of LagosLagos, Nigeria; ^2^O.N. Allen Laboratory for Soil Microbiology, Department of Soil Science, University of Wisconsin–Madison, MadisonWI, USA

**Keywords:** sediment, PAH, hydrocarbons, microbial diversity, Illumina, Africa, estuary, Lagos lagoon

## Abstract

Estuarine sediments are significant repositories of anthropogenic contaminants, and thus knowledge of the impacts of pollution upon microbial communities in these environments is important to understand potential effects on estuaries as a whole. The Lagos lagoon (Nigeria) is one of Africa’s largest estuarine ecosystems, and is impacted by hydrocarbon pollutants and other industrial and municipal wastes. The goal of this study was to elucidate microbial community structure in Lagos lagoon sediments to identify groups that may be adversely affected by pollution, and those that may serve as degraders of environmental contaminants, especially polycyclic aromatic hydrocarbons (PAHs). Sediment samples were collected from sites that ranged in types and levels of anthropogenic impacts. The sediments were characterized for a range of physicochemical properties, and microbial community structure was determined by Illumina sequencing of the 16S rRNA genes. Microbial diversity (species richness and evenness) in the Apapa and Eledu sediments was reduced compared to that of the Ofin site, and communities of both of the former two were dominated by a single operational taxonomic unit (OTU) assigned to the family *Helicobacteraceae* (Epsilonproteobacteria). In the Ofin community, Epsilonproteobacteria were minor constituents, while the major groups were Cyanobacteria, Bacteroidetes, and Firmicutes, which were all minor in the Apapa and Eledu sediments. Sediment oxygen demand (SOD), a broad indicator of contamination, was identified by multivariate analyses as strongly correlated with variation in alpha diversity. Environmental variables that explained beta diversity patterns included SOD, as well as levels of naphthalene, acenaphthylene, cobalt, cadmium, total organic matter, or nitrate. Of 582 OTU identified, abundance of 167 was significantly correlated (false discovery rate *q*≤ 0.05) to environmental variables. The largest group of OTU correlated with PAH levels were PAH/hydrocarbon-degrading genera of the *Oceanospirillales* order (Gammaproteobacteria), which were most abundant in the hydrocarbon-contaminated Apapa sediment. Similar *Oceanospirillales* taxa are responsive to marine oil spills and thus may present a unifying theme in marine microbiology as bacteria adapted for degradation of high hydrocarbon loads, and may represent a potential means for intrinsic remediation in the case of the Lagos lagoon sediments.

## Introduction

Estuaries are important components of the global biosphere and play a variety of roles that range from providing habitat for a diversity of plants and animals, to the creation of unique biogeochemical zones that support key transformations in nutrient cycles. However, estuaries also often have significant exposure to anthropogenic activities and thus become polluted with a wide range of organic and inorganic compounds carried in agricultural, industrial, and municipal wastes (Chapman et al., 2013; Elliott and Elliott, 2013; Floehr et al., 2013; Chakraborty et al., 2014; Brady et al., 2015; Li and Duan, 2015; Tappin and Millward, 2015; Machado et al., 2016; Ribeiro et al., 2016). Estuarine sedimets are the ultimate repositories of these contaminants, and thus the in-depth knowledge of the impacts of contaminants upon sediment biology is an important prerequisite to understanding the broader effects on estuaries as a whole.

Sediments house a wide variety of benthic organisms, but prokaryotic microorganisms are particularly key in carrying out biogeochemical processes that are essential in natural nutrient cycling as well as in the fate and behavior of pollutant compounds. Sediments are unique microbial habitats in which a variety of aerobic and anaerobic processes can occur in different redox zones (Hunter et al., 2006; Crump et al., 2007; Zeng et al., 2011; Oni et al., 2015). The co-existence of these activities is important in natural biogeochemical cycling (e.g., linking methane production and consumption) as well as in affecting the fate of pollutants. For example, polycyclic aromatic hydrocarbons (PAHs) are widespread pollutants of sediments (Patel et al., 2013; Johnston and Leff, 2015; Louvado et al., 2015; Revathy et al., 2015; Waigi et al., 2015; Xia et al., 2015) and while anaerobic transformations of some PAH are known, aerobic processes tend to be more effective (Doyle et al., 2008; Fernandez-Luqueno et al., 2011). Conversely, halogenated organic compounds such as polychlorinated biphenyls (PCBs) and trichloroethylene are relatively recalcitrant to degradation by aerobic processes, but have significant potential for transformation by anaerobes (i.e., those of Chloroflexi class Dehalococcoidia) that dehalogenate these compounds as part of a respiratory process (Zanaroli et al., 2015; Matturro et al., 2016). Thus, an understanding about the impacts of pollutants upon sediment microbial communities should include consideration of effects on groups important in natural transformations, as well as those that may be active in biodegradation processes.

Lagos lagoon, located in Lagos State, southwestern Nigeria is one of Africa’s largest estuarine ecosystems. It receives loads from four large rivers (Yewa, Ogun, Ona, and Osun), which collectively drain more than 103,626 km^2^ of Nigeria. The estuary is urbanized, and borders the densely populated city of Lagos from which a large amount of wastewater is released into the lagoon daily. The lagoon is also industrialized, and is the location of Africa’s largest port, as well as the location of extensive petroleum tank farms (Obafemi, 2008). Other areas of the lagoon boarder the forest belt, and are less intensively impact by urban and industrial activities (Alani et al., 2012; Nsikak et al., 2014; Adama et al., 2015; Amaeze et al., 2015). Thus, Lagos lagoon has areas that range from severely impacted by anthropogenic activities to those having comparatively low levels of direct impact. The potential effect that the varied types and intensities of anthropogenic impacts have had on the microbial communities in this ecosystem has not been explored. The goal of the present study was to fill that knowledge gap, and elucidate the structure of microbial communities in sediments of Lagos lagoon that varied in levels and types of anthropogenic impacts, with a view to identify microbes important in natural biogeochemical cycles that may be adversely affected by pollution, and those that may serve as degraders of environmental contaminants, especially PAHs.

## Materials and Methods

### Study Sites and Sampling

**FIGURE 1:**
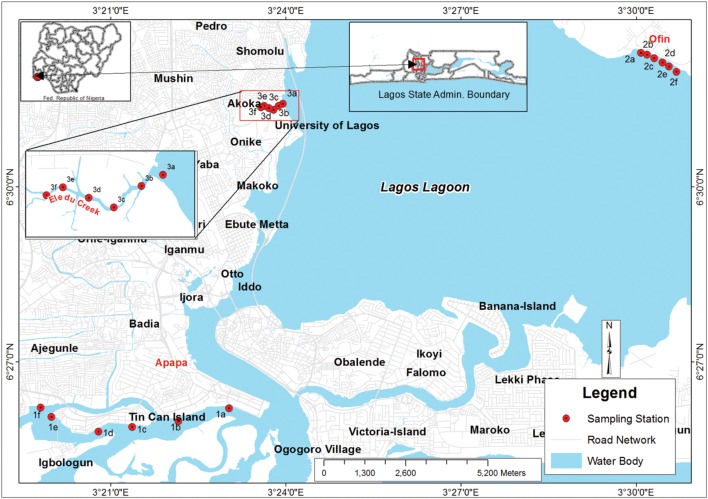
**Geographical map of the Lagos lagoon showing the sampling sites and stations.** The sampling sites are given in red text: Apapa, Ofin, and Eledu. Expanded detail of the Eledu site is given in the inset.

Lagos lagoon is a tropical, coastal estuary that stretches from Cotonou in the Republic of Benin, and extends to the fringes of the Niger Delta in Nigeria along its 257 km course (located between Latitude 6°26′ 12.48 to 6°31′57″ and Longitude 3°19′ 48″ to 3°30′ 41″). Sediments from three sites were examined (**Figure 1**). The Apapa and Eledu sites were located adjacent to zones of dense population and municipal waste discharge. Additionally, the Apapa site was near Tin Can Island, which is the site of the largest port in West Africa and houses a high density of petroleum tank farms. The Ofin site lacked a major population on the adjacent shoreline and local industrial effluent was limited to a textile industry.

A 20 kg Ven Veen Grab (KC Research Equipment, Silkeborg, Denmark) sampler for collecting undisturbed bottom sediments was used in collecting sediments from six stations at each site. Samples were pooled in to a sterile aluminum foil pans and transported on ice to the lab and stored at -20°C prior to chemical analysis and nucleic acid extraction. The sampling strategy adopted was similar to other studies examining biogeography of microbial communities (Fierer and Jackson, 2006; Roesch et al., 2007; Bates et al., 2011, 2013) and employed compositing of sediment samples by site to focus on comparisons between sites rather than within sites. Physicochemical parameters including pH determined with pH meter (model M501Rev Jenway CE350EU), total organic matter (TOM) and total phosphorus (TP) were determined as described previously (Froelich et al., 1979; Chopra and Kanwar, 1998) and sediment oxygen demand (SOD) was determined as described by Prince (Prince, 2015). Nitrate and chemical oxygen demand (COD) were measured according to Radojević and Bashkin (2006). For heavy metal analysis, sediment samples were dried at 80°C for 48 h, then gently ground with a rolling pin to disaggregate the sample then sieved to collect particles <63 μm. The size-fractionated samples (2.0 g) were digested with a solution of concentrated HNO_3_ (0.3 ml) and HCl (6.0 ml) to near dryness and allowed to cool before 20 ml of 5.0 M HNO_3_ was added (Tsai et al., 2003). The solutions were allowed to stand overnight and filtered. The filtrates were transferred into a 100 ml volumetric flask and made up to the mark with 0.5 M HNO_3_ (Binning and Baird, 2001). Heavy metals present in the filtrate were determined by using flame atomic absorption spectrophotometry. PAH analysis was done by USEPA method 8270D. Sediment samples (10 g) were sonically extracted with methylene chloride. The extracts were then dried, concentrated, and exchanged into cyclohexane. Sample clean up was done by passage over silica gel. The purified extracts were then exchanged back to methylene chloride and analyzed by gas chromatography mass spectrometry. Extract composition was examined by total ion scans, and the USEPA 16 priority pollutant PAH were identified and quantified by using primary and secondary ions.

### DNA Extraction, PCR Amplification and Sequencing

Methods for microbial community analysis were done by following protocols described previously (De Gannes et al., 2015). Microbial DNA from the sediment samples was isolated by using the UltraClean Soil DNA (MoBio Laboratories) extraction kit and quantified by fluorometry using a Qubit^®^ 3.0 (ThermoFisher Scientific) with the dsDNA broad range protocol. To minimize variability from the DNA extraction process, 12 extractions were prepared from each sediment in batches of 0.25 g, which were pooled to generate three 1 g-equivalent samples. A 1 μl aliquot from each of the 1 g-equivalent extracts was used for generation of three amplicon libraries for each site. The libraries were created with universal prokaryotic primers 515F and 806R (barcoded) that targeted the V4–V5 region of the 16S rRNA gene (Caporaso et al., 2010). The PCR conditions used were: denaturing for 3 min at 94°C, 35 cycles at 94°C for 45s, 50°C for 60s, and 72°C for 90 s, followed by 10 min of final primer extension at 72°C. PCR was done in 25 μl reaction mixtures; 13 μl of PCR grade H_2_O (MoBio Laboratories), 5 Primer Hot master mix, 0.5 μl of 10 μM of each primer and 1.0 μl of DNA template. All amplification was done in three replicate 25 μl PCR reactions. Following initial amplification, library size was verified on an Agilent DNA 1000 chip, and cleaned using a 1X volume of Mag PCR clean-up beads (Axygen Biosciences, Union City, CA, USA). Following PCR, samples were cleaned and normalized by using a Sequal Prep Normalization Plate (Life Technologies, Carlsbad, CA, USA). Quantity and quality of the libraries were assessed using an Agilent DNA 1000 chip and Qubit^®^ dsDNA High Sensitivity Assay Kit, respectively, and were standardized to 2 nM prior to pooling and sequencing. Sequencing was done with an Illumina Miseq system (Illumina, San Diego, CA, USA) by using Miseq reagent kit v.3 (Illumina) to generate 2 bp × 300 bp reads at the University of Wisconsin Biotechnology Center Madison, Wisconsin, USA. Images were analyzed using the standard Illumina Pipeline, version 1.8.2.

### Sequence Data Processing and Analyses

Data analysis was done by the University of Wisconsin–Madison, Biotechnology Center. Illumina datasets were de-multiplexed by using MiSeq Reporter v. 2.2.31 (Illumina) with a Q20 minimum value as a quality filter, Reads were adapter- and quality-trimmed by using the Skewer trimming program (Jiang et al., 2014). Flash was used to merge paired end reads into amplicons (Magoč and Salzberg, 2011), which were then quality filtered. QIIME v. 1.9.1 (Caporaso et al., 2010) analysis used an open-reference OTU picking process: amplicon sequences were first clustered against a reference sequence collection (Greengenes v. 2013_08) using 97% similarity. Sequences that did not align with the reference sequence collection were clustered *de novo* at 97% similarity. Taxonomic assignment of each OTU was established by using the RDP Classifier (Wang et al., 2007). Sequence alignments were filtered to remove variable regions prior to phylogenetic tree creation. Singleton OTUs and OTUs that could not be aligned by using PyNAST were removed from the data set. For alpha diversity analysis (Chao1 and Shannon metrics), data from all three replicates for each sample was pooled and rarefaction applied using an upper limit that corresponded to the size of the smallest library (33,729 quality-filtered amplicons (**Supplementary Figure S1**)). Prism 6 (Graphpad, La Jolla, CA, USA) was used to display the composition of each library.

### Statistical Analyses

Univariate linear regression of OTU abundance against environmental parameters was done by using the R programming environment^1^ False discovery rates were determined by the method of Benjamini and Hochberg (1995) and significance assessed by setting the resulting *q* values ≥ 0.05. The results of the linear regression were compiled in Excel to generate heat maps, wherein environmental parameters that were significantly correlated with the abundance of an OTU were cells highlighted in orange, and those lacking correlation were colored a background grey. The OTUs were organized by taxa (phyla), and thus the heat maps were used to display the patterns of environmental correlations across phyla. Multivariate analyses were done by using Primer-E v. 6 (PRIMER-E Ltd, Lutton, UK). For biological data (Illumina libraries) the numbers of quality-filtered amplicons assigned to a given OTU were normalized to the total quality-filtered amplicons assigned in a given library. The data set was square root transformed to down weight effects of highly abundant OTU and then a resemblance matrix constricted based on Bray-Curtis similarities (Clarke et al., 2008). Beta diversity patterns (among-sample similarities in microbial community structure) were examined by principal coordinate analysis (PCA) and results displayed by ordination. Similarity contours overlaid on PCA ordinations were based on groupings developed with the CLUSTER routine. Relationships between beta diversity patterns and environmental data were examined by using two non-parametric tests, the RELATE and the Bioenvironmental Step (BEST) routines. RELATE is a Mantel-type test that generates a Spearman Rho test statistic that measures congruence between matrices of biotic or abiotic data. BEST identified subsets of physicochemical variables that gave rank order similarities (Euclidean distance) between sediments that best matched the rank order Bray-Curtis similarities of microbial community composition (Clarke et al., 2008). Multivariate analysis was also done to examine the results of linear regression analysis to elucidate potential patterns in combinations of environmental variables that correlated with OTU abundance. For these tests, environmental variables were transformed to binary data (1 or 0) based on whether or not they showed a significant correlation to OTU abundance. Thus, for any given OTU, a variable that was significantly correlated with abundance was scored as “1”, and any lacking a significant correlation were scored as “0”. The data was then used to construct a resemblance matrix based on Euclidean distance, and CLUSTER analysis applied. The results were displayed as dendrograms, which illustrated groupings of environmental variables that tended to co-occur as significantly related to abundance of OTU. The output of the CLUSTER analysis was also applied to the heatmaps, and the environmental variables were listed in the tables in the same groups and order that were displayed in the dendrograms. The goal of this approach was to present the data in way that potentially conveyed information about the relationships between environmental variables and taxonomic identities of the OTU, rather than using an arbitrary listing of environmental parameters (e.g., alphabetical, by chemical type, *etc*.).

### Accession Numbers

The Illumina sequence data reported here have been deposited in the NCBI Sequence Read Archive^2^ under accession number SRP069095.

## Results

### Sediment Characteristics

Out of the sixteen PAHs analyzed in the sediments, four composed the major fraction of these compounds: phenanthrene, fluoranthene, benzo(*b*)fluoranthene, and pyrene (**Table 1**). Total PAH levels decreased in the order Apapa > Ofin > Eledu. Eight heavy metals were determined, with the most abundant being (exclusive of iron) Zn, Cu, Ni, and Cr (**Table 1**). Total metal concentrations in the sediments decreased in the order Apapa > Ofin > Eledu. The three sites were highly dissimilar in levels of TOM, SOD, and COD, with the Apapa and Eledu sites having TOM and SOD levels at least twice that of the Ofin sediment (**Table 1**). A qualitative analysis of the sediments for halogenated organic compounds also revealed the presence of trichloroethylene in the Eledu sediment; halogenated organic compounds were not detected in the other two sites.

**Table 1:** Sediment physicochemical characteristics^a^.

	Sediment		
	
	Ofin	Apapa	Eledu
	**PAH (ug/kg)**		
	
Acenaphthene	20 ± 0.6	38 ± 0.8	5 ± 0.6
Acenaphthylene	115 ± 0.5	150 ± 0.3	60 ± 0.2
Anthracene	28 ± 0.1	64 ± 0.1	19 ± 2.1
Benzo(*a*)anthracene	261 ± 0.4	376 ± 7.7	308 ± 11.2
Benzo(*a*)pyrene	84 ± 6.8	334 ± 8.5	80 ± 0.3
Benzo(*b*)fluoranthene	492 ± 12.3	787 ± 6.6	426 ± 0.5
Benzo(*g,h,i*)perylene	231 ± 1.9	270 ± 1.5	68.6 ± 4.5
Benzo(*k*)fluoranthene	149 ± 7.9	208 ± 0.3.7	118 ± 10.1
Chrysene	196 ± 9.9	210 ± 16.1	176 ± 2.2
Dibenzo(*a,h*)anthracene	29 ± 7.9	43 ± 7.9	26 ± 7.9
Fluoranthene	531 ± 1.1	829 ± 8.4	411 ± 7.7
Fluorene	26 ± 3.5	75 ± 8.1	5 ± 1.01
Indeno(1,2,3-*cd*)pyrene	294 ± 1.2	517 ± 3.8	122 ± 5.2
Naphthalene	29 ± 3.1	35 ± 1.1	20 ± 1.1
Phenanthrene	802 ± 15.9	986 ± 12.3	710 ± 6.5
Pyrene	688 ± 12.1	719 ± 7.2	457 ± 15.1
Sum of all PAH	3,947	5,606	2,991
	
	**Heavy metals (mg/kg)**		
	
Cd	4.82 ± 0.02	4.06 ± 0.01	3.02 ± 0.03
Co	4.17 ± 0.1	4.14 ± 0.1	2.46 ± 0.4
Cr	10.43 ± 0.03	13.16 ± 0.02	8.36 ± 0.06
Cu	15.63 ± 0.57	16.96 ± 0.01	14.86 ± 0.04
Ni	15.11 ± 0.02	16.39 ± 0.21	12.16 ± 0.02
Pb	0.13 ± 0.01	0.14 ± 0.05	0.13 ± 0.01
Zn	16.73 ± 0.45	17.06 ± 0.20	13.63 ± 0.30
Sum of all metals	67.02	71.90	54.62
	
	**Other parameters**		
	
pH	8.4 ± 0.18	7.2 ± 0.2	7.8 ± 0.1
Total organic matter (g/kg)	149 ± 1.1	342 ± 0.5	428 ± 3.3
COD (mg/l)	480 ± 0.02	218 ± 0.01	1274 ± 0.1
SOD (mg/m^2^/d)	4.02 ± 0.1	12.79 ± 0.1	20.48 ± 0.1
Nitrate (mg/l)	57.3 ± 0.2	63.3 ± 0.15	70 ± 0.06
Total phosphate (mg/kg)	1.0 ± 0.2	1.62 ± 0.06	0.4 ± 0.2
Trichloroethylene^b^	Negative	Negative	Positive
Fe (mg/kg)	104.74 ± 0.57	143.74 ± 0.03	659.31 ± 0.09


*^a^For all measures, *n* = 3. Abbreviations: COD, Chemical oxygen demand. SOD, Sediment oxygen demand. ^b^Qualitative analysis.*

### Alpha- and Beta-diversity Characteristics

Alpha diversity parameters (OTU richness and evenness) differed greatly between the sediments (**Table 2**). Eledu was lowest in both OTU richness and evenness while Ofin was the highest in both of these categories. Alpha diversity of the Apapa sediment was intermediate between the Ofin and Eledu.

**Table 2:** Characteristics of Illumina libraries and alpha diversity metrics of sediment communities.

	Sediment		
	
	Apapa	Ofin	Eledu
Number of reads	131,448	118,455	111,165
Number of quality filtered amplicons	64,403	33,729	65,868
Median amplicon length (bp)	440	443	440
Number of OTU	419	503	293
Chao1	2,791	3,069	2,173
Shannon	5.32	10.09	3.91


**FIGURE 2:**
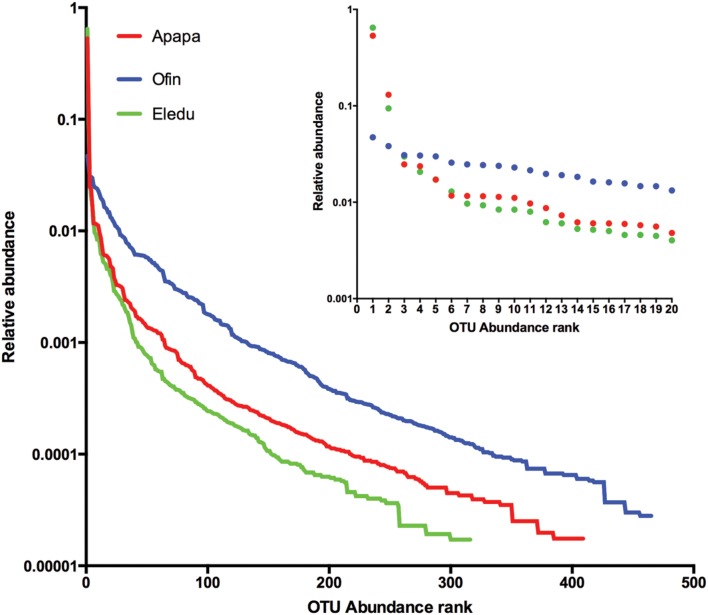
**Whitaker plots of average operational taxonomic unit (OTU) abundance in the sediment communities.** For each sediment, OTU are plotted from most abundant (rank 1) to least. The large plot shows the entire data set for each sediment, while the inset shows a detailed view of the top 20 most abundant OTU in each sediment.

Alpha diversity characteristics of the microbial communities were visualized with Whittaker plots, where line slopes reflect species (OTU) evenness and line lengths indicate OTU richness. Whittaker plots of the Apapa and Eledu communities were similar in displaying lines that were initially steeply sloped, reflecting a single dominant OTU that accounted for more than 50% of the reads in each of the libraries (**Figure 2**). The Apapa and Eledu samples were also similar in having a second highly abundant OTU. In contrast, dominant OTUs were absent from the Ofin sediment, and OTU abundance was comparatively evenly distributed (**Figure 2**). The Ofin sample also had the greatest OTU richness as evidenced by the longest sample line, followed by the Eledu and Apapa (**Figure 2**).

Identification of the environmental variables that were potential drivers of variation in microbial alpha diversity was explored by multivariate analyses. First, examination by a RELATE test yielded a Rho value of 0.395 and *p* = 0.031. The BEST routine identified the best fit between the among-sample patterns of the diversity measures with that of the environmental variables, and identified only variation in SOD levels as a strong predictor of microbial diversity (ρ = 0.894, *p* = 0.003), with both the Shannon and Chao1 metrics decreasing with increasing SOD.

**FIGURE 3:**
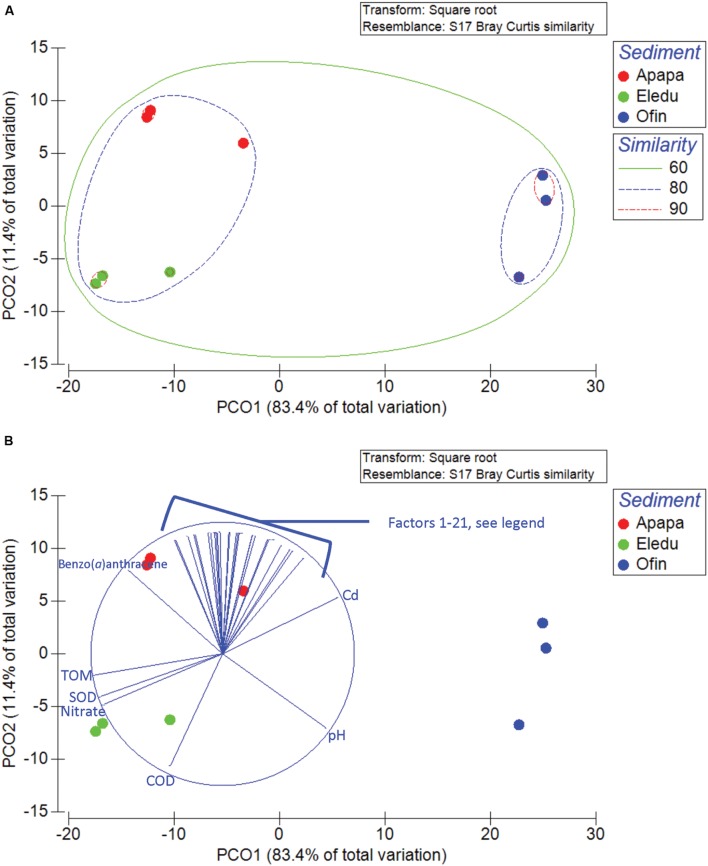
**Principal component ordinations of the sediment microbial communities showing levels of similarity among sampling sites **(A)** and environmental variables associated with the segregation of communities along the PCO axes **(B)**.** Environmental variables contained within the brackets are (left to right in the cluster): 1. Pb, 2. Benzo(*a*)pyrene, 3. Benzo(*b*)fluoranthene, 4. Benzo(*g,h,i*)perylene, 5. Cr, 6. Acenaphthene, 7. Cu, 8. Acenaphthylene, 9. Anthracene, 10. Benzo(*k*)fluoranthene, 11. Naphthalene, 12. Phenanthrene, 13. Indeno(1,2,3-*c,d*)pyrene, 14. Pyrene, 15. Chrysene, 16. Ni, 17. Dibenzo(*a,h*)anthracene, 18. Fluoranthene, 19. Fluorene, 20. Pyrene, 21. Co.

Beta diversity patterns of the sediment communities (among-sample differences in OTU composition) were examined by PCA ordination (**Figures 3A,B**). The first axis (PCO1) accounted for the majority of the variance (83%) and separated the Ofin community from those of the Apapa and Eledu sites. The Apapa and Eledu communities were separated by PCO2, which accounted for 11% of the variance. The Apapa and Eledu sediments were 80% similar to each other in microbial community composition but only 60% similar to the Ofin sediment community (**Figure 3A**). The sediment characteristics strongly associated with PCO1 were TOM, SOD, and nitrate while environmental vectors associated with PCO2 were PAH and metals (**Figure 3B**). The RELATE test yielded a relatively strong correlation between the sediment microbial community beta diversity and environmental parameters (ρ = 0.651, *p* = 0.003). The BEST routine was then employed to identify environmental variables that best explained variation in the beta diversity patterns. The ten correlations obtained identified either only one or two variables, but all yielded the same test statistic and significance (ρ = 0.878, *p* = 0.001). Factors that could individually explain beta diversity patterns were levels of Co, Cd or nitrate. The latter two variables also occurred in correlations combined with naphthalene or acenaphthylene. Two other variables, SOD and TOM, also occurred in correlations combined with naphthalene or acenaphthylene.

### Microbial Community Composition

**FIGURE 4:**
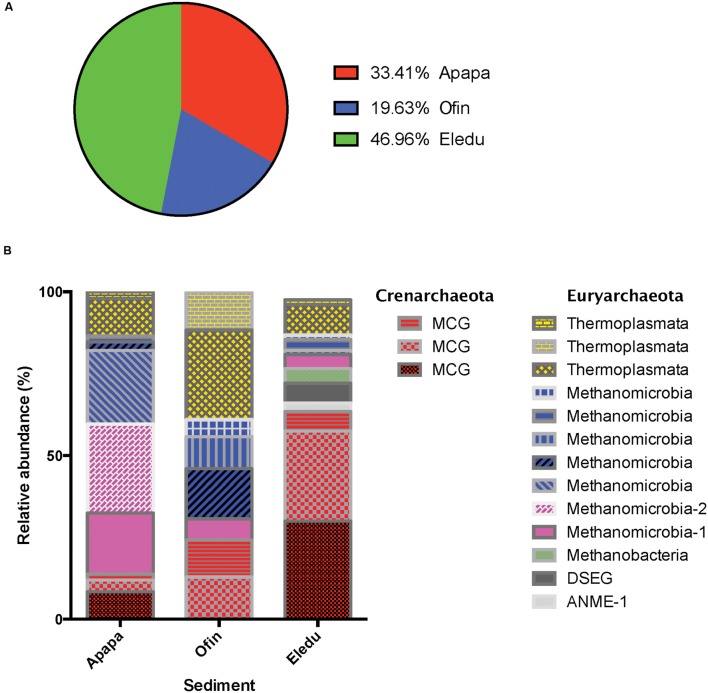
**Details of the archaeal community characteristics in the sediments.** **(A)** Relative abundance of archaeal quality-filtered amplicons in each of the sediments as a percentage of the total archaeal quality-filtered amplicons across all libraries. **(B)** Relative abundance of major archaeal taxa in each of the sediment communities as a percentage of all archaeal quality-filtered amplicons in the indicated sediment. Abbreviations are: MCG, Miscellaneous Crenarchaeaotal Group; ANME-1, Anaerobic Methanotrophic-1; DSEG, Deep Sea Euryarchaeotal Group.

Across all libraries, a total of 565 bacterial OTU (97% of quality-filtered amplicons) and 17 archaeal OTUs (3% of quality-filtered amplicons) were identified (Supplementary Table S1). Archaea were divided between two phyla, the Crenarchaeota and Euryarchaeota. None of the archaeal OTUs was assigned to the phylum Thaumarchaeota. The relative abundance of Archaea at the Eledu site was greater than that in either the Apapa or Ofin sediments (**Figure 4A**). Each site had distinct archaeal community profiles (**Figure 4B**). Eledu was unique in being dominated by the Miscellaneous Crenarchaeotal Group [MCG; now reclassified to a new archaeal phylum, Bathyarchaeota (Lloyd, 2015)], which comprised 64% of archaeal OTUs in that sediment. Apapa and Eledu sediments were similar in that archaeal communities were primarily *Euryarchaeota.* However, the types of euryarchaeotal taxa present in the Apapa and Eledu sites differed markedly. In the Apapa community, the largest euryarchaeotal group was acetoclastic methanogens of the *Methanosarcinales*, while in the Ofin sediment, acetoclastic methanogens were a relatively minor constituent and the archaeal community was composed primarily of hydrogenotrophic or methylotrophic methanogens.

**FIGURE 5:**
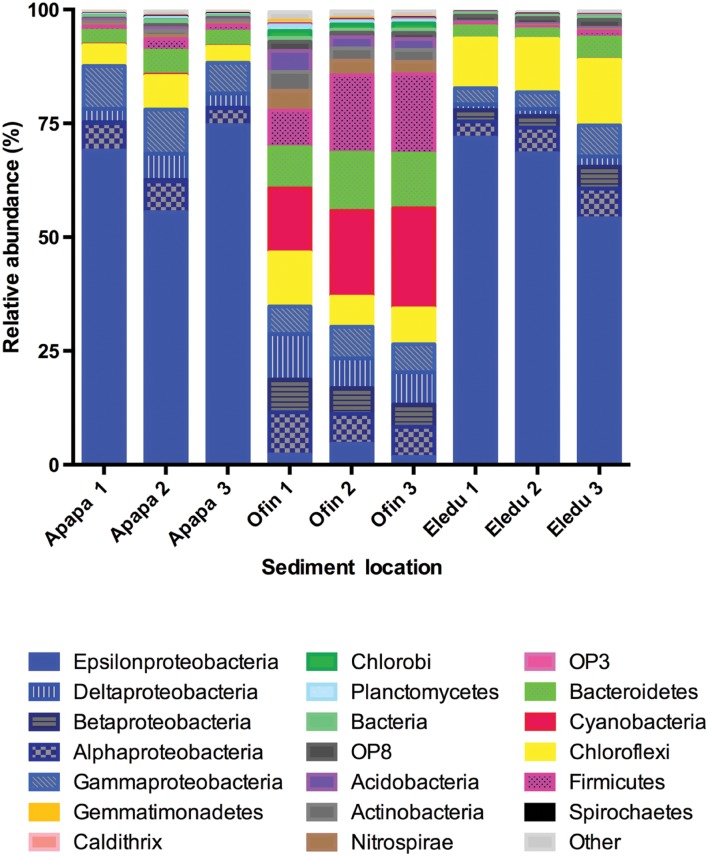
**Relative abundance of major bacterial phyla in Lagos Lagoon sediments.** The phlyum Proteobacteria is subdivided by class (all color-coded blue).

Proteobacteria predominated the microbial communities in the Apapa and Eledu sites representing 75–85% of the reads (**Figure 5**). In contrast, in the Ofin sediment, Proteobacteria comprised 30–35% of the libraries and three other phyla comprised large segments of the communities: Cyanobacteria (14–22%; excludes chloroplast sequences), Bacteroidetes (9–12%), and Firmicutes (8–17%). Other phyla that showed increased abundance in the Ofin sediment relative to the Apapa and Eledu sites were: Nitrospirae, Actinobacteria, Acidobacteria, and Chlorobi. Chloroflexi was a significant phylum in all sediment communities (**Figure 5**), but comprised the largest share of library in the Eledu sediment (13%).

In the Apapa and Eledu sediments, the dominance of Proteo- bacteria was attributable to a single OTU of the family *Helicob- acteraceae* in the Epsilonproteobacteria class (Supplementary Table S1). The microbial community composition of the Ofin sediment was divergent from that of the other sites as the Epsilonproteobacteria were relatively minor (<1%). In the Eledu samples, the dominant *Helicobacteraceae* OTU accounted for an average of 65% of the reads, and was identified as *Sulfuricurvum.* In Apapa, the dominant *Helicobacteraceae* OTU (identified only to family level) accounted for an average of 53% of the reads, and a second *Helicobacteraceae* OTU (assigned to the genus *Sulfurimonas*) comprised an additional 13% of the sequences. In the Apapa and Eledu samples, all other OTU were of comparatively low abundance (**Figure 2**). For example, in the Apapa libraries, there were only eight OTUs comprising ≥1% of the libraries while Eledu had only four OTUs with ≥1% relative abundance. In contrast, the Ofin sediment was not dominated by any individual OTU (**Figure 2**), and the single most abundant of these was a Cyanobacteria sequence that on average comprised 4% of the libraries.

In the Ofin sediment, Dehalococcoidia accounted for 21% of all Chloroflexi reads. In comparison, 31% of Chloroflexi reads were assigned as Dehalococcoidia in the Apapa samples, while Dehalococcoidia represented 84% of the Eledu Chloroflexi. Thus, there was an apparent enrichment of Dehalococcoidia in the Eledu sediment relative to the other two sites. Notably, the increased abundance of Dehalococcoidia in the Eledu sediment corresponded to the presence of trichloroethylene at that site, which was not detected in either of the other two sediments.

Six different *Oceanospirillales* genera accounted for 3% of all gammaproteobacterial sequences in the Apapa sediment, but the three main *Oceanospirillales* OTU were identified as the genera *Marinobacterium*, *Marinomonas*, and *Oleibacter*, all of which have been implicated in the biodegradation of PAH or other hydrocarbons (Teramoto et al., 2011; Dong et al., 2014). The *Vibrionales* OTU identified were assigned to the genus *Pseudoalteromonas* or to the family *Pseudoalteromonadaceae*, and were detected only in the Apapa sediment. Other known PAH/hydrocarbon degraders were *Alcanivorox* (Luan et al., 2014; Campos et al., 2015) and *Halomonas* (Gasperotti et al., 2015; Gutierrez et al., 2015). In the Eledu sediment, there was only one *Oceanospirillales* OTU (*Oleibacter*) and in the Ofin site there were no OTU identified as *Oceanospirillales*. While the Eledu and Ofin communities contained comparatively few of these key gamaproteobacterial OTU, other hydrocarbon genera were present especially from the Alphaproteobacteria (*Rhizobium, Rhodobacter*) and Betaproteobacteria (*Oxalobacteriaceae, Methylophilaceae*), which were either absent or of low abundance in the Apapa site.

**FIGURE 6:**
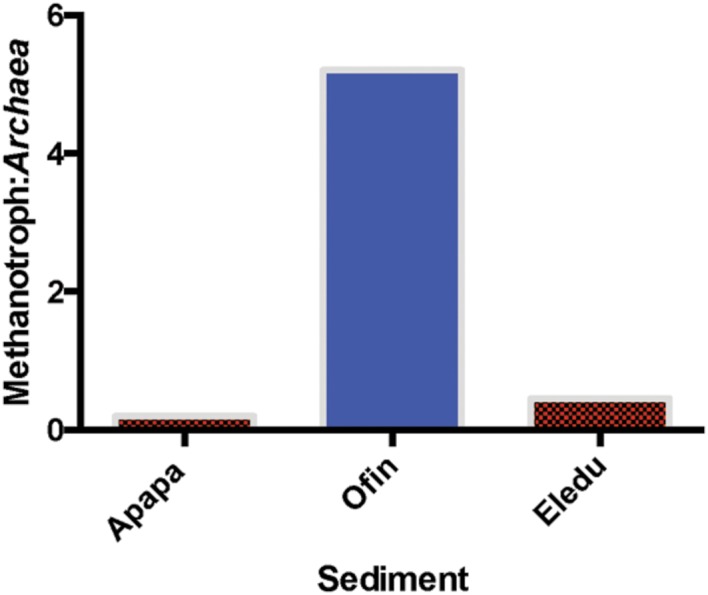
**Ratio of gammaproteobacterial methantroph quality-filtered amplicons to all archaeal quality-filtered amplicons in each sediment**.

In the Lagos lagoon a total of four OTUs were identified as alphaproteobacterial methanotrophs of the *Methylocystaceae* family, with three assigned to the genera *Methylopila*, *Methylosinus*, or *Pleomorphomonas*. Ten OTUs were assigned as gammaproteobacterial methanotrophs of the *Methylococcaceae* family identified to the genera *Methylocaldum, Methylomicrobium, Methylomonas*, and *Methylosarcina*. While both groups of methanotrophs were present at all sites, they were by far the most abundant in the Ofin sediment where they comprised 82% of all reads assigned as alphaproteobacterial methanotrophs, and 78% of all sequences identified as gammaproteobacterial methanotrophs. Furthermore, at the Ofin site, the ratio of all methanotroph reads to those of all archaeal sequences was at least ten times greater than that in communities of the Apapa or Eledu sites (**Figure 6**). Thus, there was an apparent enrichment of methanotrophs in the Ofin sediment, which may have reflected enhanced methanogenic capacity of the archaeal community in that site compared to that occurring in either the Apapa or Eledu sediments.

### Relation of OTU Abundance to Sediment Physicochemical Characteristics

A total of 167 OTUs showed significant correlations (*q*⋅FDR ≤ 0.05) in abundance to environmental variables (Supplementary Table S2). There were 50 OTUs with a single correlation (mostly to TOM), and one OTU was correlated to 21 variables (Supplementary Table S3). Sediment TOM content was the environmental variable most frequently associated with OTUs abundance (90 OTUs) with SOD the second most frequently correlated factor (Supplementary Table S4). Other environmental factors that were frequently correlated with OTU abundance were dibenzo(*a*,*h*)anthracene, benzo(*a*)anthracene, benzo(*b*)fluoranthence, benzo(*a*)pyrene and Pb (Supplementary Table S4).

**FIGURE 7:**
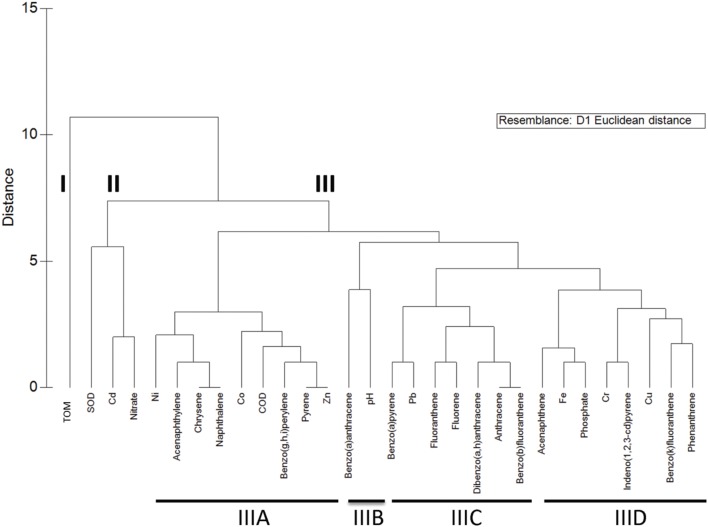
**Dendrogram illustrating groupings of environmental characteristics that frequently co-occurred as variables significantly correlated with OTU abundance.** Three main branches are indicated by I-III, and four sub-branches within branch III are marked IIIA-D.

Multivariate analyses was applied to identify potential patterns in the frequency with which sediment physicochemical characteristics co-occurred as factors correlated with OTU abundance, and a dendrogram displaying results of that analysis had three main branches (**Figure 6**). Branch I contained the dominant environmental factor, TOM, alone. Branch II included SOD as well as nitrate and Cu. Branch III was represented predominantly by PAHs and metals. Within branch III, there were four subclusters (branches IIIA-D) of PAH and/or metals that frequently co-occurred as factors correlated with OTU abundance (**Figure 7**).

**FIGURE 8:**
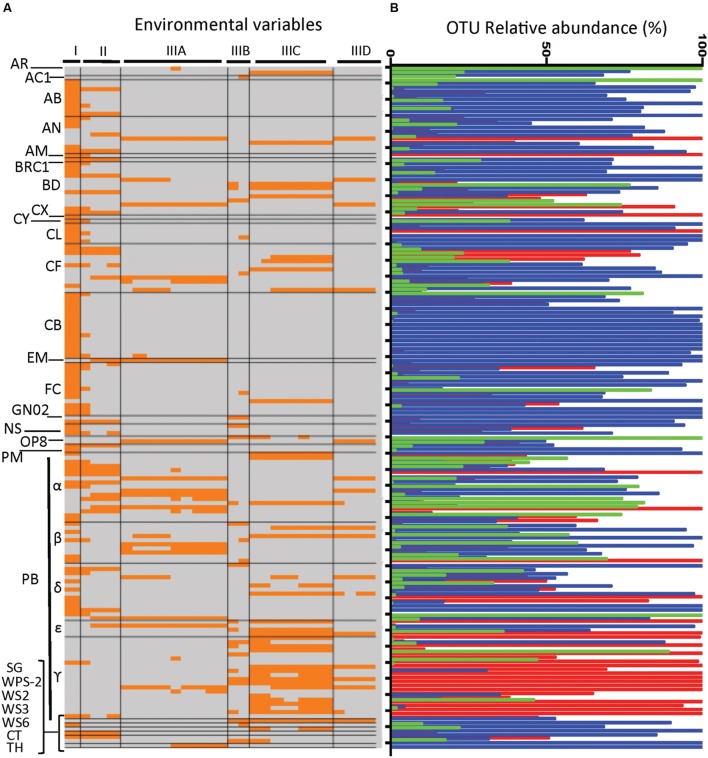
**(A)** Heat map comparing environmental variables correlated with OTU abundance as a function of OTU taxonomic identity. **(B)** Comparison of OTUs taxonomic identity with OTU abundance across sites. **(A)** Environmental factors are given across the top as the groupings determined by CLUSTER analysis (see groupings in dendrogram, **Figure 7**). The OTUs are listed along the left and are grouped alphabetically by taxa. Each line is an OTU, and sections of the line that are colored orange identify environmental variables that were significantly correlated with abundance of the OTU; line sections colored gray correspond to variables that were not significantly correlated with OTU abundance. Taxa identifications are abbreviated as: AR, Archaea; AB, Acidobacteria; AC1, candidate phylum, AN, Actinobacteria; AM, Armatimonadetes; BD, Bacteroidetes; CX, Calithrix; CY, Chlamydiae*;* CB, Cyanonbacteria; CF, Chloroflexi; EM, Elusimicrobia; FC, Firmicutes; GN02, candidate phylum; NS, Nitrospira; OP8, candidate phylum; PM, Planktomycetes; PB, Protoebacteria; CL, Chlorobi; BRC1, candidate phylum; SG, Synergistetes; WPS-2, WS2, candidate phylum; WS3, candidate phylum; WS6, candidate phylum; CT, Caldithrix; TH, Thermi. Greek letters denote proteobacterial classes: α, alpha; β, beta; γ, gamma; δ, delta, and ε, epsilon. **(B)** Relative abundance of each OTU (each line from **A**) in each of the sediments is indicated by the bars in **(B)**, which are colored by site: Apapa, red; Ofin, blue; Eledu, green. Abundance of a given OTU at each site is expressed as a percentage of the total number of quality-filtered amplicons assigned to that OTU across all sites.

**FIGURE 9:**
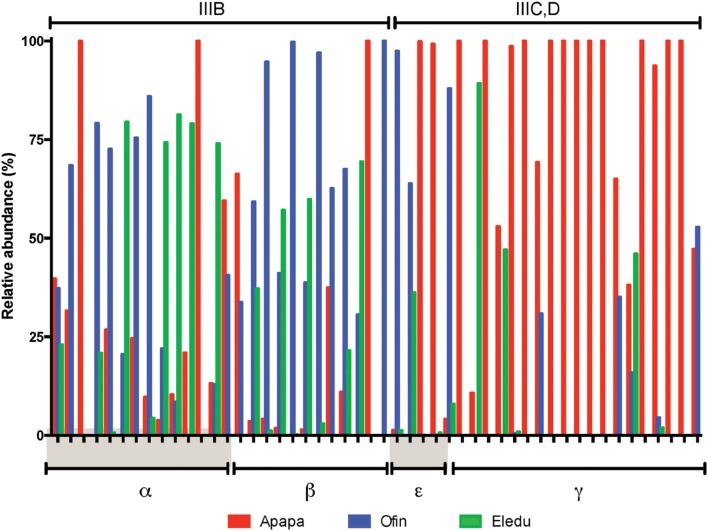
**Expanded view of proteobacterial OTU showing significant correlations in abundance to PAH/metals of subcluster III (see **Figures 7** and **8A,B**).** Bars show the relative abundance of each OTU in the indicated sediment, expressed as percentage of the total abundance of the OTU across all samples. Proteobacterial subclasses illustrated are α, alpha; β, beta; γ, gamma, and ε, epsilon. Abundance of each OTU in each sediment is in each of the sediments indicated by bar color (Apapa, red; Ofin, blue; Eledu, green) and expressed as a percentage of the total number of quality-filtered amplicons assigned to that OTU across all sediments.

The OTUs that were correlated to factors in those branches showed in some cases distinct distribution patterns in terms of both taxonomy and site. For example, the abundance of Gammaproteobacteria OTU was correlated primarily to factors in Branch IIIC (six PAHs and Pb) and to lesser extent those of branch IIID (**Figure 8A**). Furthermore, the majority of Gammaproteobacteria OTU correlated to the branch IIIC factors were most abundant in the Apapa sediment (**Figures 8B** and **9**), and many of those OTUs were identified as genera in the order *Oceanospirillales* associated with PAH/hydrocarbon degradation (discussed above). The Alphaproteobacteria and Betaproteobacteria also showed a relatively high frequency of correlations to PAH and metals, but primarily to those of branch IIIA (**Figure 8A**). In contrast to the Gammaproteobacteria, most of these Alphaproteobacteria and Betaproteobacteria OTUs were from the Ofin or Eledu sites and comparatively few were derived from the Apapa sediment (**Figures 8B** and **9**). The Epsilonproteobacteria, including the dominant *Helicobacteraceae* OTUs from the Apapa site, were also correlated with PAH and metals (branches IIIA, C, D). Compared to other proteobacterial classes, the Deltaproteobacteria had relatively few correlations to metals and PAHs, but instead were most frequently correlated with TOM (**Figure 8A**). Other taxa, such as the Acidobacteria, Chlorobi, Cyanobacteria, and Firmicutes, displayed a pattern similar to that of the Deltaproteobacteria, and had extensive correlations to TOM, but few or no correlations to metals or PAH (**Figure 8A**). These taxa were in general most abundant in the Ofin sediment (**Figure 8B**).

## Discussion

Estuarine sediments are significant repositories of pollutants worldwide (Chapman et al., 2013; Elliott and Elliott, 2013; Floehr et al., 2013; Chakraborty et al., 2014; Brady et al., 2015; Li and Duan, 2015; Tappin and Millward, 2015; Machado et al., 2016; Ribeiro et al., 2016), an in-depth knowledge of the impacts of pollutants on microbial community structure is essential to gain insights into processes that may affect the fate of pollutants specifically and biogeochemical cycling more broadly. In the Lagos Lagoon, PAHs and heavy metal pollutants were spread throughout the sediments, but concentrations of both were highest in proximity to heavily industrialized port area of Apapa. However, the Eledu sediment was also contaminated by trichloroethylene, which was not present at either of the other sites. While SOD reflects the sum of biological and biochemical processes consuming oxygen, biological decomposition of organic matter is generally the major contributor and elevated SOD values are commonly measured in sediments impacted by organic wastes (Miskewitz and Uchrin, 2013; Fields et al., 2014; Rucinski et al., 2014; Azzoni et al., 2015). The elevated SOD of the Apapa and Eledu sites was thus a strong indictor that these sediments were impacted by organic materials to a level far greater than that of the Ofin site. An impact of organic wastes on the Apapa and Eledu sediments would also be consistent with the much higher levels of TOM at these two sites compared to the Ofin site. A key environmental effect of elevated SOD is a heightened drive to hypoxia in the water column and anaerobic conditions in the sediment, the latter of which would be consistent with dominance of anaerobic Epsilonproteobacteria in the Apapa and Eledu microbial communities, bacteria that were comparatively minor constituents of the Ofin sediment.

Potential impacts of anthropogenic pollutants upon microbial communities in the lagoon sediments were reflected in variations in alpha diversity. Compared to the Ofin site, the Apapa and Eledu communities had significantly decreased species (OTU) richness as well as decreased species evenness. Furthermore, the results of the RELATE and BEST tests collectively demonstrated that differences in alpha diversity were correlated with variations in environmental parameters, with differences in SOD the most significant. Other surveys of microbial communities in marine sediments have also detected decreases in species richness (Quero et al., 2015; Fuentes et al., 2016; Wang et al., 2016) which may be linked to reduction in either the variety of ecosystem services supported by these communities or in the redundancy of microbial processes, rendering the ecosystems less resilient to perturbations.

In the present study, the impact of sediment contamination on reduction of species evenness was perhaps even more striking, with both the Apapa and Eledu communities dominated by a single OTU, *Helicobacteraceae.* Quero and coworkers also reported a single *Helicobacteraceae* OTU dominated sediments contaminated by complex mixtures of PAHs, PCBs and heavy metals (Quero et al., 2015). The apparent enrichment of *Helicobacteraceae* was consistent with a trend observed for the Epsilonproteobacteria more broadly, which are now proving to be highly abundant in wide range of anoxic environments impacted by hydrocarbons including aquifers, soil, sludge and oil reservoirs (Kasai et al., 2005; Zhang et al., 2005; Hubert et al., 2012; Keller et al., 2015). However, hydrocarbon metabolism has not been demonstrated for *Helicobacteraceae* or other Epsilonproteobacteria, thus the physiological characteristics that enable their dominance of environments such as the highly polluted areas of Lagos lagoon (as indicated by SOD) remain to be determined. One possibility is that the Epsilonproteobacteria grow as syntrophs with bacteria that metabolize hydrocarbons (Hubert et al., 2012), a role that is supported by a recent study using stable isotope probing with [^13^C_2_]-acetate of an anerobic community consuming benzene (Starke et al., 2016).

Potential effects of sediment contamination were also apparent in microbial community structure. The RELATE and BEST tests demonstrated that differences among sites in composition were correlated with variations in environmental parameters, which were best explained by organic and inorganic contaminants. The composition of the Apapa and Eledu communities was similar in the dominance of the Epsilonproteobacteria, which resulted largely from a single *Helicobacteraceae* OTU (as discussed above). In contrast, in the Ofin sediment, Epsilonproteobacteria were minor constituents, and the community was composed primarily of other proteobacterial classes, as well as Cyanobacteria, Bacteroidetes, Firmicutes, which were all comparatively minor constituents of the Apapa and Eledu communities. Thus, there were significant alterations in microbial community structure associated with sediment contamination that could lead to shifts in pathways of fundamental biogeochemical processes. For example, the Cyanobacteria represented a potentially significant pathway for nitrogen uptake *via* biological nitrogen fixation (Howarth et al., 1988; Karlson et al., 2015; Adam et al., 2016), which was effectively eliminated in the contaminated sites. Also, the classes of Bacteroidetes and Firmicutes identified in the Ofin sediment have well established activities such as organic matter decomposition and fermentation that are keystone processes in anaerobic food webs (Hunter et al., 2006; Crump et al., 2007; Zeng et al., 2011; Oni et al., 2015). The absence or diminution of these groups in the Apapa and Eledu communities could thus have consequent effects on the mechanisms and pathways of carbon and H_2_ flow.

Alterations in carbon cycling resulting from sediment contamination could also have been reflected in the archaeal communities that impact methane flux. Archaea were least abundant in the Ofin site, but the community was composed predominantly of hydrogenotrophic and methylotrophic methanogens, representing a significant potential for methane production. Notably, the Ofin site displayed evidence of a strong enrichment in methanotrophic bacteria, which would be consistent with enhanced methane formation. The Eledu site harbored the largest archaeal community, which was dominated by the crenarchaeotal MCG class that may, or may not, be involved in either production or consumption of methane (Kubo et al., 2012; Evans et al., 2015; Chen and He, 2016). The Apapa sediment harbored a relatively large archaeal community that was distinguished by a dominance of acetoclastic methanogens, possibly reflecting limiting H_2_ levels (Beckmann et al., 2011; Beckmann and Manefield, 2014; Lv et al., 2015). While the exact impact that these variations in archaeal community structure may have on the levels of methane production is unknown, fundamental physiological differences between these groups would probably result in differences in methane flux.

A goal of this study was to gain insights into the microbial communities that may mediate PAH biodegradation *in situ* in sediments of Lagos lagoon, and there were strong parallels in the types of potential hydrocarbon degraders apparently enriched in the lagoon with those identified in other marine ecosystems impacted by point source hydrocarbon spills (Hazen et al., 2010; Dubinsky et al., 2013). The massive oil release from the *Deepwater Horizon* has been the subject of field studies utilizing deep sequencing analyses (Illumina or pyrosequencing) and provided a database of marine bacteria that are responsive to hydrocarbon exposure, at the heart of which are Gammaproteobacteria of the *Oceanospirillales* and *Vibrionales* orders (Dubinsky et al., 2013; Gutierrez et al., 2013; King et al., 2015). Moreover, hydrocarbon metabolism has been demonstrated for these taxa in laboratory experiments by *in situ* labeling with stable isotopes and/or physiological characterization of pure cultures (Gutierrez et al., 2013). In the present study, the same gammaproteobacterial genera implicated as key PAH/hydrocarbon degraders responsive to large oil spills formed an important core of potential hydrocarbon-degraders that were enriched in the Apapa sediment, which had the highest levels of PAHs contamination. In contrast, in the sediments less heavily impacted by PAHs, the genera of potential hydrocarbon degraders shifted to the Alphaproteobacteria and Betaproteobacteria. Thus, Proteobacteria were a significant group potentially affiliated with PAH, but the classes varied depending upon levels of PAHs and perhaps other factors. In future studies, approaches such as stable isotope probing (SIP) combined with meta-omics (genome, transcriptome, and/or proteome) could be applied to investigate the functions of organisms that have been hypothesized from the present study. For example, as noted above, SIP *via* [^13^C_2_]-acetate pulsing has recently provided evidence of Epsilonproteobacteria as the predominant acetate consumers in a community of anaerobes that consumed benzene (Starke et al., 2016).

## Conclusion

This study provided insights into the potential impacts that PAHs and complex pollutants may have upon the sediment community in the Lagos lagoon, Nigeria. The communities of two sites strongly impacted by organic wastes (as reflected in elevated SOD) were significantly different from that of a comparatively non-impacted site, and exhibited diminution of OTUs involved in key biogeochemical processes such as nitrogen fixation and methane metabolism. The contaminated sites were also similar in that, although waste streams of differing composition impacted each site, communities of both sediments were dominated by one or two species of *Helicobacteraceae*, which were insignificant in the non-impacted site. The apparent enrichment of *Helicobacteraceae* in heavily polluted sites was consistent with findings of prior investigators, and highlighted a need to understand the physiological characteristics of *Helicobacteraceae* that enable effective colonization of such ecosystems. Lastly, data from this project indicated that hydrocarbon/PAH degrading genera of the order *Oceanospirillales* where potentially important in the sediment most heavily impacted by hydrocarbon pollution, which was consistent with findings from studies of marine oil spills. Thus, the *Oceanospirillales* appear to present a unifying theme in marine microbiology as adapted for degradation of high hydrocarbon loads in general, and present a potential means for intrinsic remediation in the case of the Lagos lagoon sediments in particular.

## Author Contributions

Experimental design and sample collection (CO, SA, EU, MI, and OA), Sample analysis (CO, SA, EU, MI, OA, and WH), Data analysis (CO, SA, EU, MI, OA, and WH), manuscript preparation (CO, SA, OA, and WH).

## Conflict of Interest Statement

The authors declare that the research was conducted in the absence of any commercial or financial relationships that could be construed as a potential conflict of interest.

## Footnotes

1 www.R-project.org.

2 http://www.ncbi.nlm.nih.gov/sra

## Supplementary Material

**FIGURE S1 — Rarefaction analyses of entire libraries for the indicated parameters: observed species (A), Chao1 metric (B), and Shannon metric (C).** Note: for comparisons between sites of Chao1 and Shannon metrics, rarefaction was done to a common maximum number of sequences, which corresponded to the size of the smallest library (Ofin, 33,729 quality-filtered amplicons).
