# Supplementary figures and images for: Microbial Communities in Sediments of Lagos Lagoon, Nigeria: Elucidation of Community Structure and Potential Impacts of Contamination by Municipal and Industrial Wastes
Source: Front Microbiol. 2016 Aug 5;7:1213. doi: 10.3389/fmicb.2016.01213 (PMC4974257; doi:10.3389/fmicb.2016.01213)

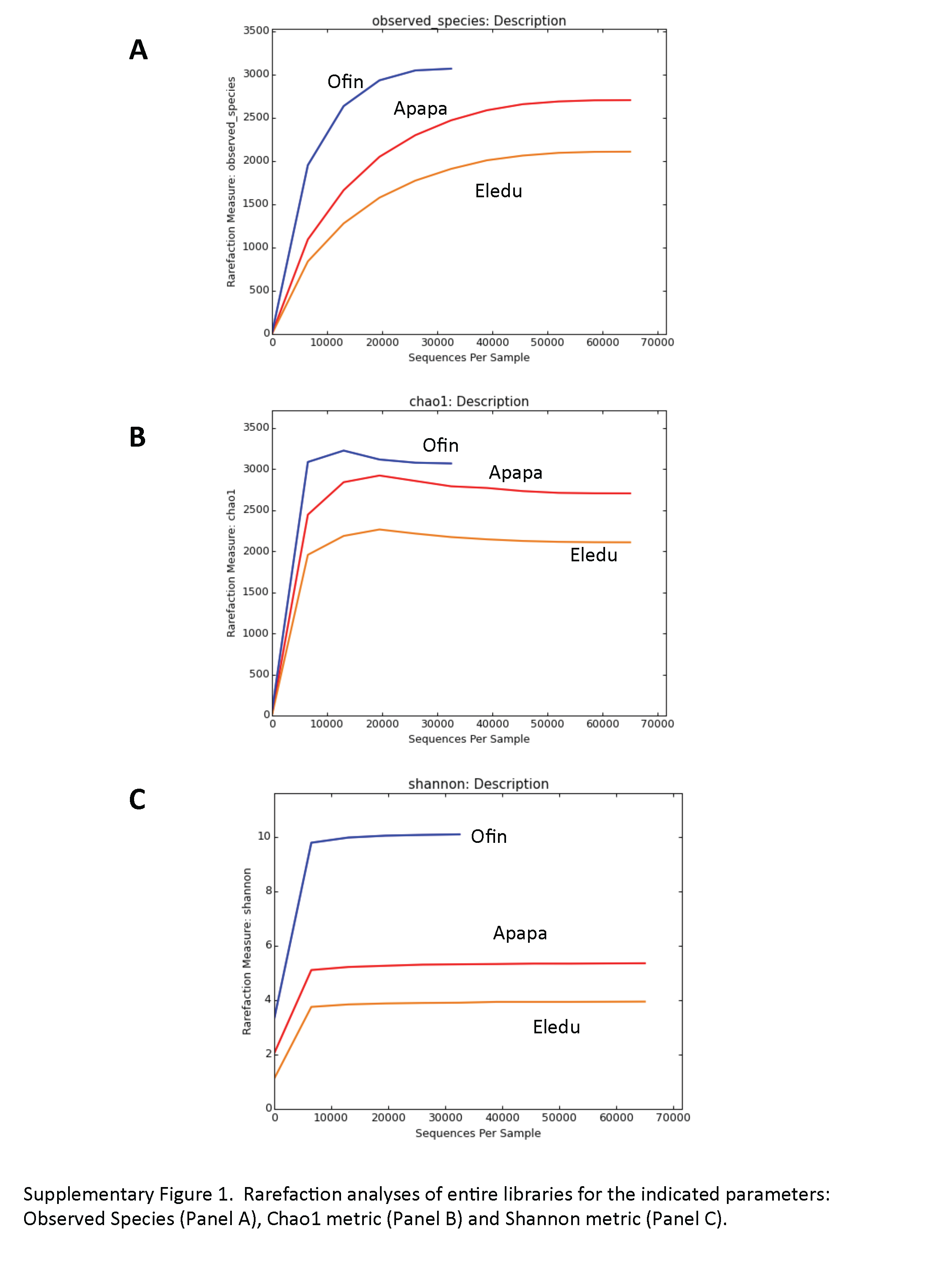

Supplement: Supplementary file 1 [file Image_1.TIFF]
